# Nitrate sensing by the maize root apex transition zone: a merged transcriptomic and proteomic survey

**DOI:** 10.1093/jxb/erv165

**Published:** 2015-04-23

**Authors:** Sara Trevisan, Alessandro Manoli, Laura Ravazzolo, Alessandro Botton, Micaela Pivato, Antonio Masi, Silvia Quaggiotti

**Affiliations:** ^1^Department of Agriculture, Food, Natural Resources, Animals and Environment, University of Padova, Agripolis, Viale dell’Università, 16, 35020 Legnaro (PD), Italy; ^2^Proteomics Centre of Padova University, VIMM and Padova University Hospital, Via Giuseppe Orus, 2, 35129 Padova, Italy

**Keywords:** iTRAQ, nitrate, nitric oxide, RNA-Seq, root transition zone, *Zea mays* L.

## Abstract

Nitrate is an essential nutrient for plants, and crops depend on its availability for growth and development, but its presence in agricultural soils is far from stable. In order to overcome nitrate fluctuations in soil, plants have developed adaptive mechanisms allowing them to grow despite changes in external nitrate availability. Nitrate can act as both nutrient and signal, regulating global gene expression in plants, and the root tip has been proposed as the sensory organ. A set of genome-wide studies has demonstrated several nitrate-regulated genes in the roots of many plants, although only a few studies have been carried out on distinct root zones. To unravel new details of the transcriptomic and proteomic responses to nitrate availability in a major food crop, a double untargeted approach was conducted on a transition zone-enriched root portion of maize seedlings subjected to differing nitrate supplies. The results highlighted a complex transcriptomic and proteomic reprogramming that occurs in response to nitrate, emphasizing the role of this root zone in sensing and transducing nitrate signal. Our findings indicated a relationship of nitrate with biosynthesis and signalling of several phytohormones, such as auxin, strigolactones, and brassinosteroids. Moreover, the already hypothesized involvement of nitric oxide in the early response to nitrate was confirmed with the use of nitric oxide inhibitors. Our results also suggested that cytoskeleton activation and cell wall modification occurred in response to nitrate provision in the transition zone.

## Highlight

A combined untargeted approach was adopted to achieve a picture of the transcriptional and proteomic profiles typifying the maize root transition zone in response to nitrate.

## Introduction

Nitrogen (N) is one of the most important minerals affecting plant growth, development, and production. In aerobic soils, nitrate is the major source of N for most plant species (Wang YY et al., 2012). Its concentration in the soil fluctuates in time and space (Barber, 1995); thus, when soil N is lacking, N fertilizer applications are used to sustain crop cultivation (Hirel *et al.*, 2007; Robertson and Vitousek, 2009). The incorporation of N into crops is relatively inefficient, with only around 50% of applied N being utilized (Raun and Johnson, 1999; Hodge *et al.*, 2000; Glass, 2003). Therefore, public concerns regarding N leaching from agricultural lands to water resources have increased (Tilman *et al.*, 2002; Kant *et al.*, 2011). Exceeding nitrate concentrations in drinking water may pose risks to young animals and human health, and a potential cancer risk from nitrate and nitrite in water and food has been reported (Weitzberg and Lundberg, 2013).

Nowadays, in a context of both economic depression and severe environmental law restrictions, farmers have to limit the inputs in crops, although both high productivity and product quality are still required (Dong *et al.*, 2004). Therefore, determining how plants cope with their limited resources represents a significant and necessary challenge in order to improve N-use efficiency.

Plants respond to limiting N through a complex series of physiological, morphological, and developmental responses. A large number of studies have demonstrated that up to 10% of the *Arabidopsis* genome (Canales *et al.*, 2014) and approximately 7% of the maize transcriptome are nitrogen responsive (Yang *et al.*, 2011).

${\text{NO}}_{3}^{-}$ is known to be a dual-function molecule for many plants, being both the major N source and a signalling molecule (Wang YY et al., 2012), inducing changes in the transcriptome and proteome, and thus controlling many aspects of metabolism and development (Vidal and Gutiérrez, 2008; Ho *et al.*, 2009; Prinsi *et al.*, 2009; Krouk *et al.*, 2010*a*; Bouguyon *et al.*, 2012; Wang X et al., 2012; Vidal *et al.*, 2013). The molecular mechanisms by which plants react to nitrate fluctuations are complex and could have a great impact on root development. Cellular profiling of five *Arabidopsis* root cell types allowed the demonstration that the N-induced root developmental plasticity is highly cell specific and finely regulated within the root (Gifford *et al.*, 2008).

The organ devoted to explore regions of the soil is the root, and the root apex seems to function as a dynamic sensor of the external environment (Baluška and Mancuso, 2013). The root apex structure consists of a distinct zonation, which is comprised of a meristem and a zone of rapid cell elongation separated by a transition zone (TZ) (reviewed by Baluška *et al.*, 2010). The TZ has been demonstrated to combine endogenous (hormonal) and/or exogenous (sensorial) stimuli, functioning as a sensory centre able to re-elaborate information from the external environment in a developmental response (reviewed by Baluška *et al.*, 2010). Cells of the TZ have been demonstrated to be very sensitive to touch and extracellular calcium (Ishikawa and Evans, 1992; Baluška *et al.*, 1996), gravity (Masi *et al.*, 2015), auxin (Mugnai *et al.*, 2014), osmotic stress (Baluška and Mancuso, 2013), aluminium (Marciano *et al.*, 2010; Sivaguru *et al.*, 2013; Yang *et al.*, 2014), and oxidative stress (Mugnai *et al.*, 2014). Moreover, the TZ has received much attention in studies devoted to the action of hormones (Moubayidin *et al.*, 2013; Muraro *et al.*, 2013; Takatsuka and Umeda, 2014). Recently, Manoli *et al.* (2014) demonstrated that the control of nitric oxide (NO) homeostasis occurring in maize root after nitrate perception takes preferentially place at the level of the TZ and that this mechanism could be involved in the regulation of root growth by nitrate. A cell-sorting whole-genome approach led to the discovery of highly localized and cell-specific N responses (Gifford *et al.*, 2008), but little information about specific interactions between nitrate and cells of the TZ is currently available.

Because of the importance of maize as one of the most cultivated cereal crops worldwide, several studies, based mainly on microarray technology, have monitored the genome-wide transcriptional changes occurring in this species in response to fluctuating ${\text{NO}}_{3}^{-}$ concentrations (Liu *et al.*, 2008; Trevisan *et al.*, 2011, 2012*a*, *b*; Xu *et al.*, 2011; Zhao *et al.*, 2013; Zamboni *et al.*, 2014).

Recently, RNA sequencing (RNA-Seq) has become a useful tool to provide high-resolution and detailed information on the transcriptional regulation of genes expression (Martin *et al.*, 2013; Vidal *et al.*, 2013).

In this study, a transcriptome analysis using RNA-Seq technology was assessed to compare gene expression profiles in a TZ-enriched segment of maize root exposed to short-term nitrate treatments. To complement this study, proteome variations were also investigated using isobaric tags for relative and absolute quantitation (iTRAQ) (Wiese *et al.*, 2007), a gel-free, mass spectrometry quantitative technique.

Our results provide global evidence of the specific role played by this root domain in the nitrate response. Furthermore, an unequivocal contribution of NO to the nitrate-induced transcriptional response in the TZ is postulated. However, our findings also indicate the existence of NO-independent signalling pathways, which seem to depend both on nitrate itself or on some nitrate assimilation products other than NO.

## Materials and methods

### Growth of maize seedlings

Seeds of maize inbred line B73 were sown and then transferred, after germination, to nutrient solution as described by Manoli *et al.* (2014). In order to evaluate the expression of selected genes in four different portions of roots after nitrate provision, roots of 4-d-old seedlings were harvested after 2h of nitrate supply/depletion and the four fragments were immediately cut and frozen (−80 °C), for both the treatment (+${\text{NO}}_{3}^{-}$ ) and the negative control (–${\text{NO}}_{3}^{-}$ ). The four zones sampled were: 1, meristem-enriched zone (<3mm from the root tip); 2, TZ-enriched portion (the next 0.8cm); 3, elongation zone-enriched portion (the next 0.8cm); and 4, maturation zone (the residual portion). For RNA-Seq and proteomic analyses, zone 2 from two independent biological replicates was processed. For quantitative PCR (qPCR) analyses three or five independent replicates were utilized and the study was extended to the other three root portions (zones 1, 3, and 4) and to seedlings treated with 1mM sodium tungstate dehydrate (Na_2_WO_4_.2H_2_O) and 1mM 2-(4-carboxyphenyl)- 4,4,5,5-tetramethylimidazoline-1-oxyl-3-oxide (cPTIO) (Manoli *et al.*, 2014), to investigate further the role of NO (for details, see below).

### mRNA-Seq and bioinformatic analyses

Total RNA was extracted using TRIzol reagent (Invitrogen, San Giuliano Milanese, Italy). Poly(A) mRNAs were purified with an Agencourt AMPure XP beads kit (Beckman Coulter, Beverly, MA, USA) from 2 µg of total RNA (RNA integrity number > 7), fragmented for 3min at 94 °C, and used for library preparation with a mRNA-Seq Sample Prep kit v.2.0 (Illumina, San Diego, CA, USA). Single-read sequencing was carried out on a HiSeq2000 machine (Illumina) at the IGA Technology Services (Udine, Italy). Base calling was performed using the Illumina Pipeline and sequences were trimmed with ERNE (Vezzi *et al.*, 2012). TopHat (Trapnell *et al.*, 2012) was used to map and annotate the sequences on the B73 reference genome (ZmB73_RefGen_v2) (Schnable *et al.*, 2009) and Cufflinks software (Trapnell *et al.*, 2012) was used for the analysis of differentially expressed genes (DEGs). Cuffmerge allowed us to create a single unified assembly from each individual Cufflinks assembly. Transcripts with a false discovery rate (FDR) of ≤0.05 were taken as highly significant DEGs.

For details and additional information, see: http://ccb.jhu.edu/software/tophat/manual.shtml and http://cole-trapnell-lab.github.io/cufflinks/manual/.

### Chromosome localization of DEGs

Physical position of genes with a statistically significant differential expression *(P*≤0.01) was determined according to the coordinates listed in the GFF file available at the Phytozome database (http://www.phytozome.net/maize.php). Only genes that were included with a mobile window of 21 Mbp containing at least 10 genes were visualized in the map. Charts were generated with Microsoft Excel 2011 for Mac (v.14.4.4).

### RNA extraction, cDNA synthesis, and quantitative reverse transcription PCR (qRT-PCR) analysis

Total RNA was extracted using TRIzol reagent (Invitrogen), as described by Trevisan *et al.* (2011). Total RNA (500ng) was pre-treated with RQ1 RNAse-free DNAse (Promega, Milano, Italy) (Falchi *et al.*, 2010) and reverse transcribed to cDNA, as described by Manoli *et al.* (2014).

Primer sequences for the selected genes are listed in Supplementary Table S1 at *JXB* online. Primers were designed with the Primer3 web tool (v.0.4.0; http://frodo.wi.mit.edu/primer3/; Rozen and Skaletsky, 2000) and further verified with the PRATO web tool (Nonis *et al.*, 2011; http://prato.daapv.unipd.it).

Relative quantification of transcripts by qRT-PCR was performed in a StepOne Real-Time PCR System (Applied Biosystems, Monza, Italy) as described by Nonis *et al.* (2007). Reactions were performed using SYBR Green chemistry (Applied Biosystems), following the manufacturer’s instructions. Reverse-transcribed RNA (2.5ng) was used as template in each reaction as indicated by Trevisan *et al.* (2011). Melting-curve analysis confirmed the absence of multiple products and primer dimers. Data were exported and analysed according to the method of Livak and Schmittgen (2001) using the membrane protein PB1A10.07c (*MEP*, GRMZM2G018103) as the reference gene (Manoli *et al.*, 2012).

### Promoter analyses

*Cis*-regulatory motifs were identified in the promoter regions of selected genes using Promzea (http://promzea.org, Liseron-Monfils *et al.*, 2013). A region of 1000bp upstream from the predicted transcription start site was analysed and the common predicted motifs were compared with known promoter motifs in the AthaMap database (Bülow *et al.*, 2009) using STAMP (Mahony and Benos, 2007).

### Protein extraction and *in situ* trypsin digestion

iTRAQ analysis was carried out as described previously by Tolin *et al.* (2013), with minor changes. Total root proteins were extracted and 70 µg of protein samples was loaded onto a precast 4–12% SDS-PAGE gel. Single bands were excised, washed with 50mM triethylammoniumbicarbonate (TEAB), and dried under vacuum after dehydration with acetonitrile. Cysteines were reduced with 10mM dithiothreitol in 50mM TEAB (1h, 56 °C) and alkylated with 55mM iodoacetamide for 45min at room temperature in the dark. Gel pieces were washed with 50mM TEAB and acetonitrile and dried. Proteins were digested *in situ* with sequencing-grade modified trypsin (Promega, Madison, WI, USA) at 37 °C overnight (12.5ng μl^–1^ of trypsin in 50mM TEAB). The obtained peptides were extracted three times with 50 μl of 50% acetonitrile in water. One microgram of each sample was withdrawn and analysed by liquid chromatography tandem mass spectrometry (LC-MS/MS) to check the digestion efficiency. The remaining peptide solution was dried under vacuum.

### iTRAQ labelling, strong cation exchange peptide fractionation, and LC-MS/MS analysis

Peptides were labelled with iTRAQ reagents (AB Sciex, USA) according to the manufacturer’s instructions. Two replications for each condition were labelled with the iTRAQ tags (114 and 115 for N supplied, and 116 and 117 for N deprived samples, respectively). Samples were analysed separately by LC-MS/MS. Labelled peptides were vacuum concentrated, fractionated, and subjected to MS analyses.

The raw LC-MS/MS files were analysed using Proteome Discoverer 1.4 (Thermo Fisher Scientific), connected to a Mascot Search Engine server (Matrix Science, London, UK). The spectra were searched against a *Zea mays* L. protein database (Sun *et al.*, 2009) (http://ppdb.tc.cornell.edu/). FDRs were calculated using the Proteome Discoverer. MS/MS spectra containing fewer than five peaks or with a total ion count below 50 were excluded. Only proteins that were identified in all three independent experiments were considered. The quantification was performed normalizing the results on the median value of all measured iTRAQ reporter ratios. A fold change (relative to the control) of ≥1.3 or ≤0.77 indicated an increased or decreased protein, respectively.

### Gene/protein annotation and enrichment analyses

DEGs and proteins were annotated with Gene Ontology (GO) terms according to the annotation file available at the Phytozome database (http://www.phytozome.net/maize.php). Enrichment analyses for both transcripts and proteins were performed with the Blast2GO software (http://www.blast2go.com; Conesa *et al.*, 2005; Conesa and Götz, 2008; Götz *et al.*, 2008, 2011) using the overall annotation as a reference and with a FDR of ≤0.05.

To get an improved functional annotation, the protein domains present in the DEGs (*P*≤0.01) were clustered according to the InterPro database (Hunter *et al.*, 2012) by PLAZA v.3.0 (Proost *et al.*, 2015).

## Results

### Reads processing, transcriptome *de novo* assembly, and evaluation

RNA-Seq was used to generate the transcriptomic profiles of the early response to ${\text{NO}}_{3}^{-}$ in the selected portion of root (zone 2). Nitrate-depleted (24h) roots were provided with 1mM ${\text{NO}}_{3}^{-}$ for 2h and compared with nitrate-starved roots of the same age. After removal of low-quality and contaminated reads, RNA-Seq revealed 158 Mbp raw reads, ranging from 65 to 26 million per sample (Table 1). For each set of conditions, more than 80% of quality-evaluated reads were mapped to the maize genome sequence. Cufflinks was then used to assemble the aligned reads into transcripts and estimate their abundance to analyse differential expression (Trapnell *et al.*, 2010). A total of 109 882 transcripts was expressed in the two analyses. Among the transcripts detected [reads per kb per million (RPKM) >0)], 154 were significantly responsive to ${\text{NO}}_{3}^{-}$ (*P*≤0.01, FDR≤0.05). Another 524 transcripts were classified as differentially regulated by ${\text{NO}}_{3}^{-}$ , but their significance range was less stringent (*P*≤0.01, FDR>0.05). These groups were used to dissect the transcriptional responses associated with the nitrate treatment. Genes are listed in Supplementary Table S2 at *JXB* online. Of 154 genes (*P*≤0.01, FDR≤0.05), 111 were upregulated (72%), while 43 were downregulated (28%). Among the upregulated genes, 16 DEGs (13%) were classified as transposable elements and 34 (21%) as uncharacterized proteins.

**Table 1.** Summary of reads obtained by RNA-Seq analysis For each analysis, two biological replicates were processed (+N sample: libraries 1 and 2; –N sample: libraries 3 and 4).

Result	Libraries							
	1		2		3		4	
No. of total reads	38787677	100%	65608508	100%	26009381	100%	28335261	100%
No. of mapped reads	33537854	86%	57435252	88%	22449519	86%	23561167	83%
Unique	12117740	31%	20536079	31%	8177365	31%	8386919	30%
Multimatch	21420114	55%	36899173	56%	14272154	55%	15174248	54%
No. of reads not mapped	5249823	14%	8173256	12%	3559862	14%	4774094	17%

**Fig. 1.**
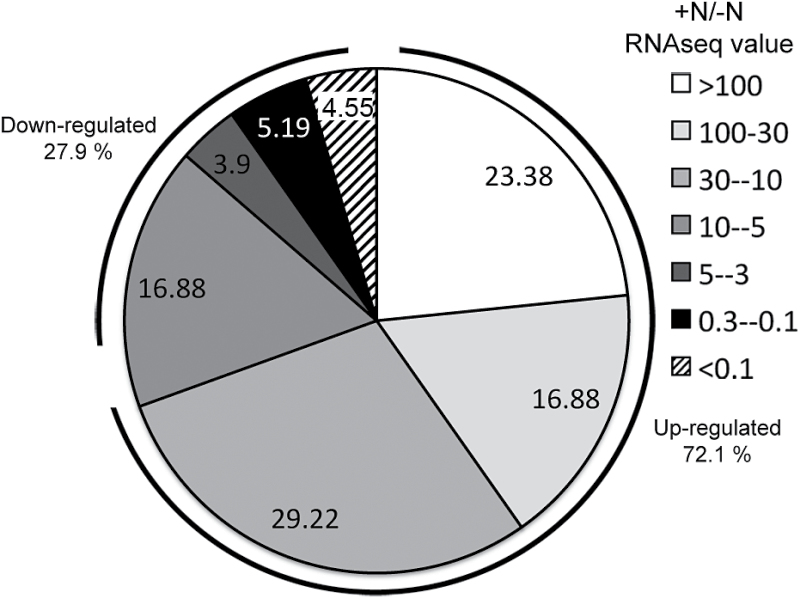
Graphic distribution of DEGs identified (FDR≤0.05) by RNA-Seq analysis from the comparison between TZ-enriched samples of nitrate-starved seedlings transferred for 2h in a nitrate-supplied (1mM ${\text{NO}}_{3}^{-}$ -) or depleted (–N, negative control) solution. DEGs were classified as upregulated (+N/–N>1) or downregulated (+N/–N<1) according to their RPKM values, and data are shown as percentages in the graph. Among the up- and downregulated groups of transcripts, several ranges of induction or repression are shown.

A considerable part of the differentially expressed transcripts had fold changes greater than 30 (8%), but the largest number of them showed a 3- to 30-fold change induction (46%) (Fig. 1). The transcription levels of 24% of the downregulated genes showed a fold change of between 0.3 and 0.1, and approximately 4% were lower than 0.1 (Fig. 1).

### Annotation and classification of DEGs into functional categories

**Fig. 2.**
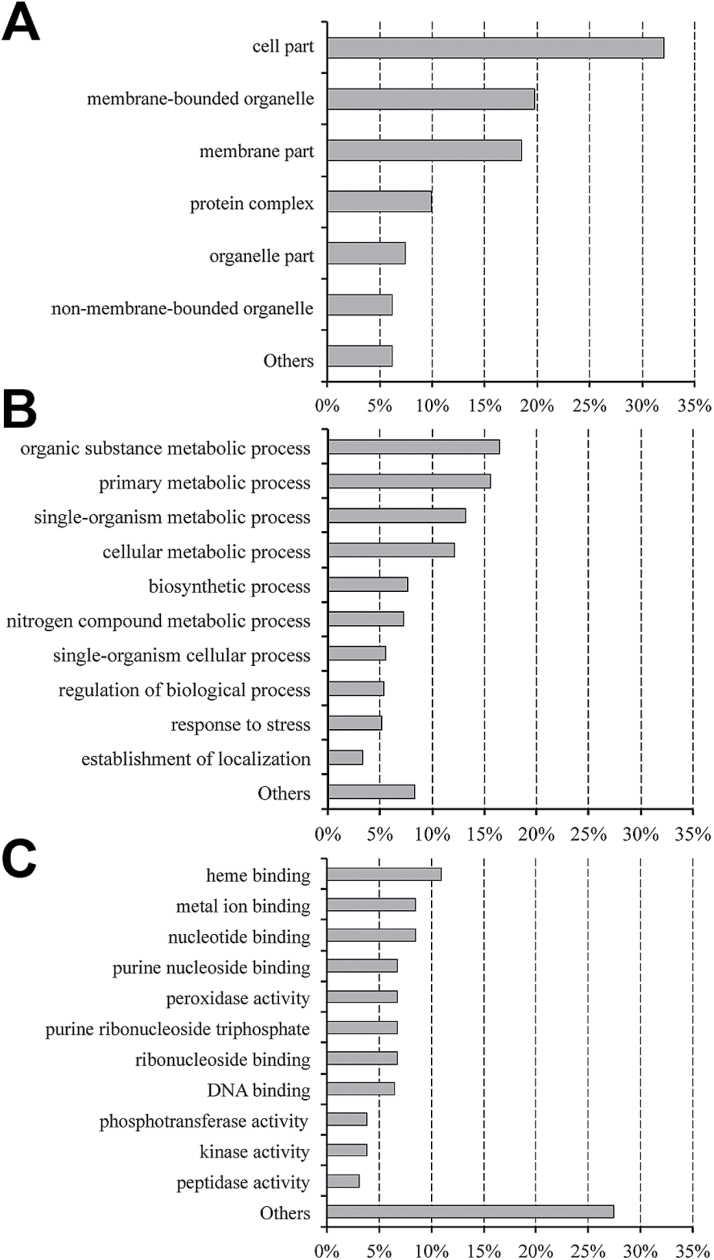
Histograms for GO (Blast2GO) classification of DEGs (*P*≤0.01) isolated by RNA-Seq analysis in the three main categories: ‘cellular component’ (A), ‘biological process’ (B) and ‘molecular function’ (C). The *x*-axis indicates the percentage of the annotation distribution in each category.

To characterize further the transcriptome response to nitrate, a GO analysis was carried out on DEGs identified by RNA-Seq (*P*≤0.01) (Fig. 2). Overall, 86% of the DEGs were successfully classified into the three main GO categories: cellular component, biological process, and molecular function. In the first category, the largest groups were ‘cell part’, ‘membrane-bounded organelle’, and ‘membrane part’. Among the biological process subcategories, ‘organic substance metabolic process’, ‘primary metabolic process’, and ‘single-organism metabolic process’ were dominant. In the third classification, the most recurrent term was ‘heme binding’, followed by ‘nucleotide binding’ and ‘metal ion binding’. Almost 7% of the DEGs were annotated as peroxidases, and 4% had a kinase activity. This classification was confirmed by domain clustering of the isolated DEGs (*P*≤0.01) according to the InterPro database (Supplementary Table S3 at *JXB* online). One or more protein domains were found in 86% of the analysed accession, resulting in a total of 1365 isolated domains. The largest groups were related to peroxidases and kinases. Several domains belonging to transcription factor protein families and DNA-binding proteins were found (CCAAT-binding transcription factor, zinc finger, basic helix–loop–helix, GRAS, MADS-box, NAC domain, homeobox domain, AP2/ERF domain, Myb domain, and Armadillo-type fold).

The remaining accessions showed interesting protein domains, such as LRR, Hsp family, ABC transporter-like, MFS, AAA+ ATPase, auxin-induced protein, Aux/IAA-ARF, LEA, LOB, DREPP, and t-SNARE. As shown by GO analysis, a large part of the protein domains identified were related to ‘oxidation–reduction process’ (oxidoreductases, peroxidases, multicopper oxidase, cytochrome P450, alcohol dehydrogenases, carotenoid oxygenases, light-dependent protochlorophyllide reductases, and uroporphyrin-III C-methyltransferases).

**Fig. 3.**
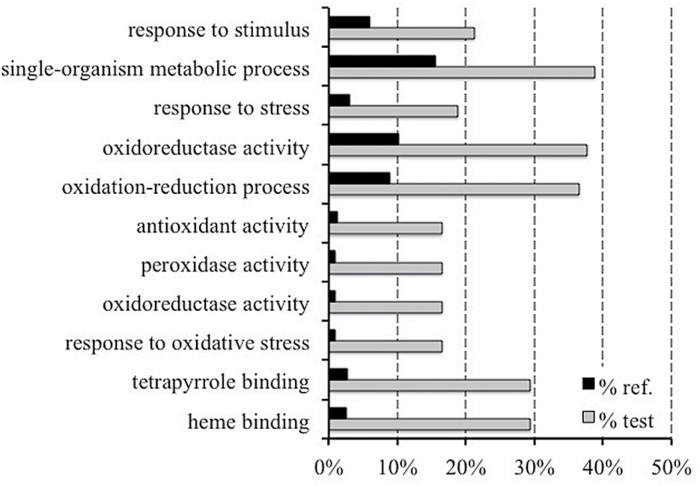
Identification of over-represented GO terms in the DEG set (FDR≤0.05) by enrichment analysis (Blast2GO). The graph represents the percentage of annotated GO (‘biological process’ and ‘molecular function’) categories of the identified data set that were found to be significantly enriched (FDR≤0.05).

An enrichment analysis was performed to discover significantly over-represented functional categories according to two major GO functional domains (biological processes and molecular function; Fig. 3). This analysis was performed only on DEGs with *P*≤0.01 and FDR≤0.05. Five GO terms related to biological processes (‘response to stress’, ‘response to oxidative stress’, ‘oxidation-reduction process’, ‘response to stimulus’, and ‘single-organism metabolic process’) were significantly over-represented among both up- and downregulated genes in response to N treatments. Considering the molecular functions, the enriched terms were ‘heme binding’, ‘tetrapyrrole binding’, ‘oxidoreductase activity’, ‘peroxidase activity’, and ‘antioxidant activity’.

### Chromosomal localization of genes responding differentially to nitrate treatment

**Fig. 4.**
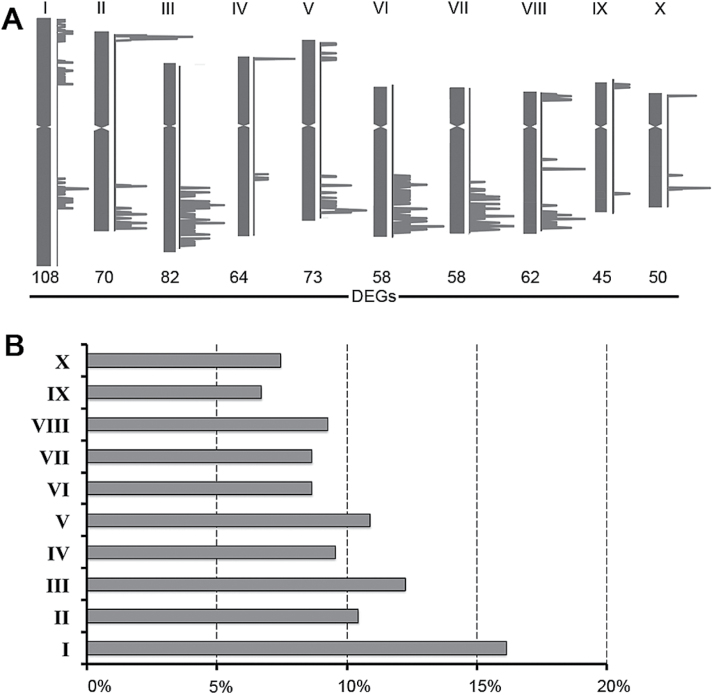
Physical position (A) and frequency (B) of DEGs (*P*≤0.01) on the 10 maize chromosomes. In (A), each chromosome is represented by a vertical grey bar, supported by graphics indicating the genetic positions of a subset of DEGs identified by RNA-Seq analysis. Only genes included within a mobile window of 21 Mbp containing more than 10 differential transcripts are shown. The number of genes in each region was then converted to a percentage of the total number of genes for the chromosome and represented as a graph in (B).

To define the genomic distribution of the DEGs, their chromosomal position was determined (Fig. 4). This analysis revealed an overall distribution of the DEGs on the 10 chromosomes, with a gene density per chromosome ranging from 6.7 to 16.1% (Fig. 4B). The highest density was observed on chromosome 1, and the lowest on chromosome 9, but chromosomes 3 and 7 had the largest clusters of DEGs.

### Validation of sequencing data by qRT-PCR

The expression levels of 41 nitrate-regulated transcripts were analysed further by qRT-PCR to examine the reliability of the observed changes between treatments. Genes were selected randomly according to both their transcription profiles and their putative functions. The transcript levels were measured on a sample obtained from roots harvested together with those used for the RNA-Seq analysis (technical repetition) and also on samples extracted from five other independent biological replicates. The list of 41 genes together with their expression profiles and the results of qRT-PCR validation are shown in Supplementary Table S4 at *JXB* online.

Since only two biological replicates were utilized for RNA-Seq, a number of both false positives and negatives was expected. Therefore, 11 of the 41 transcripts selected for validation were chosen among those that were differential but less significant (*P*≤0.01 and FDR>0.05, or *P*>0.01; for details, see Supplementary Table S2). Six transcripts belonging all to those characterized by *P*≤0.01 and FDR≤0.05 were not confirmed by qRT-PCR analysis. In contrast, all 11 selected for validation among those that were differential but not significant (*P*≤0.01, FDR>0.05) were fully confirmed by qRT-PCR analysis on five independent biological repetitions. These results suggested that the number of differential transcripts could be higher than Cufflinks analysis predictions, possibly due to the low number of biological replicates utilized for RNA-Seq.

**Fig. 5.**
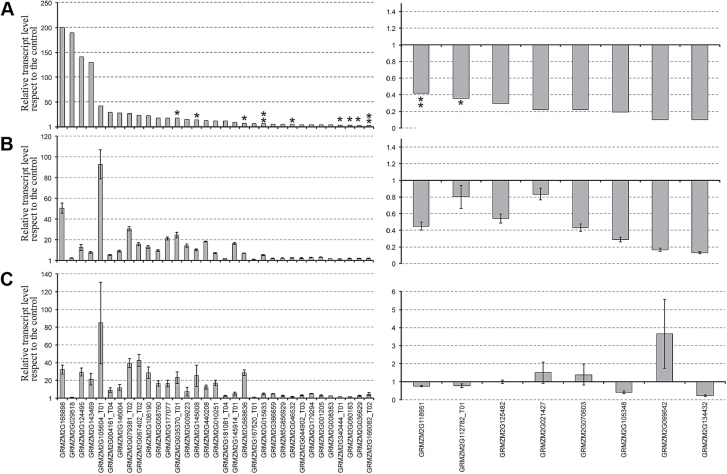
RNA-Seq profile validation. Relative expression profiles of the selected 41 genes identified from RNA-Seq analysis (A) were assessed by real-time qRT-PCR in both technical (B) and biological (C) replicates and are shown as relative expression obtained from +N/–N ratios, according to RPKM values of each DEG analysed. In (A), asterisks above columns indicate the significance of data (no asterisk, FDR≤0.05; *P≤0.01, FDR>0.05; **FDR>0.05). In (B) and (C), error bars represent the SEM of two technical replicates (B) and five biological replicates (C). Upregulated genes (+N/–N>1 relative transcript level according to RNA-Seq analysis) are shown in the left panel, while downregulated (+N/–N<1 according to RNA-Seq analysis) are shown in the right panel. RPKM detected for GRMZM2G015933 in the –N condition was arbitrarily fixed to 0.001 to calculate the +N/–N ratio.

Figure 5 shows the comparison of the transcript accumulation measured for each of the 41 genes by RNA-Seq analysis (Fig. 5A), qRT-PCR on RNA derived from the same plants used for RNA-Seq (Fig. 5B), and on other five independent biological replicates (Fig. 5C).

The set of genes chosen from the RNA-Seq output list included five previously identified genes (Trevisan *et al.*, 2011), which are expressed in the cells of the TZ in response to nitrate supply (*NR*, *HB1*, *HB2*, *NiR*, and *NRT2.1*; Manoli *et al.*, 2014). In the present study, all showed a strong induction of expression in response to nitrate provision (2h, 1mM), fully confirming our previous results and thus supporting the reliability of the experimental design adopted.

Overall, the qRT-PCR results confirmed the RNA-Seq profiles (except for six false positives), even if the entity of their fluctuations varied with respect to RNA-Seq data. This may have been due to the different sensitivity of RNA-Seq analysis and the global normalization methods utilized and/or to the low precision of the average values obtained from the two biological repetitions. Among the 35 transcripts that were validated by qRT-PCR, 31 were upregulated and only four were downregulated in response to nitrate.

### Nitrate differently affects the regulation of gene expression in the four root portions

To evaluate the specificity of the response of the maize root TZ (zone 2) to nitrate, the expression of the 35 transcripts selected previously was also assessed on the other three zones of root (zones 1, 3, and 4), both in nitrate-depleted seedlings and after 2h of nitrate provision.

The five genes encoding NRT2.1, NR, HB2, NiR, and HB1, which were within the list of 35 validated transcripts, are already known as nitrate-responsive genes specifically regulated in the TZ (Manoli *et al.*, 2014; Trevisan *et al.*, 2014). The present study fully confirmed this finding for all five genes (transcripts 25, 28, 30, 33, and 34), which showed a prominent transcriptional responsiveness to nitrate in zone 2.

**Fig. 6.**
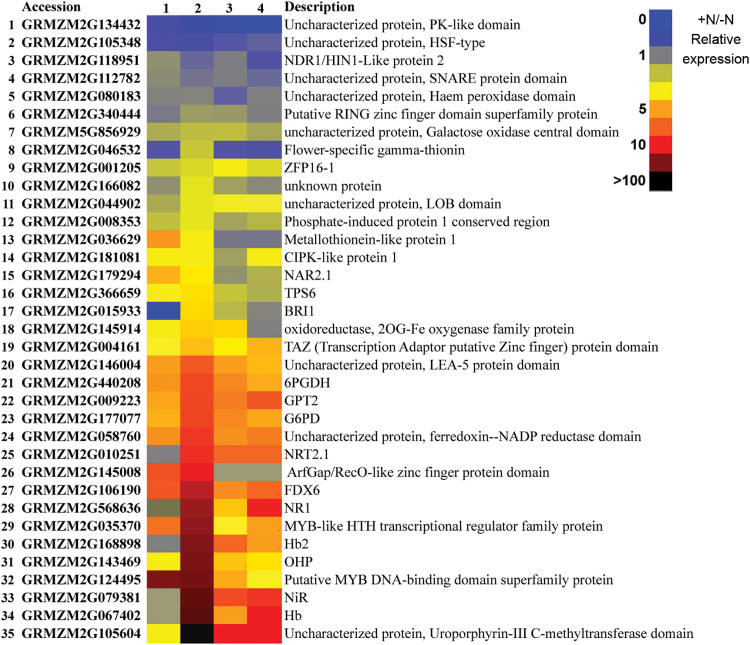
Heat map representation of qRT-PCR of differential relative expression of 35 selected DEGs in four sections (1, meristem-enriched zone; 2, TZ-enriched portion; 3, elongation zone-enriched portion; 4, maturation zone-enriched portion) of primary root seedlings. Analysis was conducted using five independent repetitions. The expression levels were normalized against the maize *MEP* gene and expression in the –N TZ samples was set as 1 using the 2^–ΔΔ*C*^_T_ method. Data for each region are reported as +N/–N qRT-PCR relative expression values. The colour bar indicates high to low expression. The responsive transcripts, together with an identifying number (1–35), are listed on the left of panel, and the function description on the right. (This figure is available in colour at *JXB* online.)

The profile of expression of all 35 genes following 2h of nitrate supply (1mM) in the four different portions of root is shown in Fig. 6. Although variable and specific profiles were detected, the majority of the upregulated genes displayed a similar behaviour, with the maximum extent of transcription induction in zone 2 (TZ-enriched) in comparison with the other three zones. A few exceptions were represented by genes showing a high induction of transcription in all four zones, thus being more transversally regulated by nitrate along the entire root (i.e. transcripts 9, 19, 20, 21, 24, and 27).

**Fig. 7.**
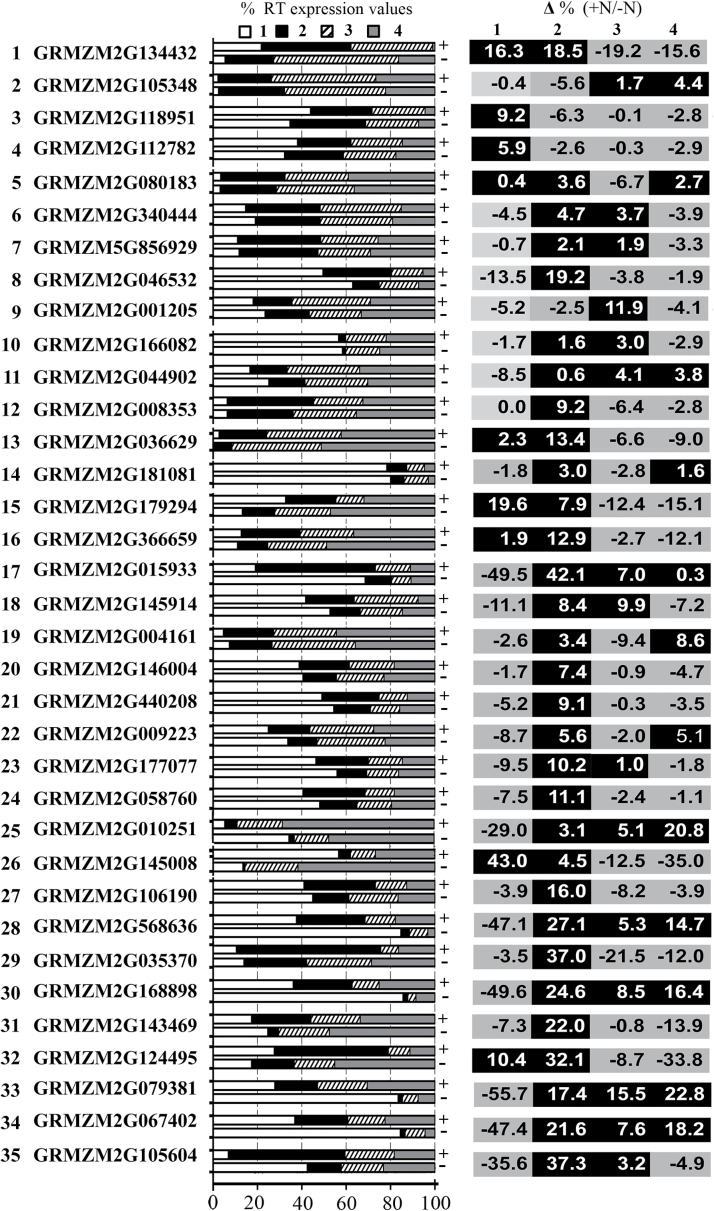
Percentage of transcript distribution in the four different root portions in nitrate-depleted (–N) and in nitrate-supplied (+N) root. Transcript abundance (%) of the 35 selected transcripts recorded in response to 2h of nitrate supply (+N) or depletion (–N) in each of the four primary root portions (1, meristem-enriched zone; 2, TZ-enriched portion; 3, elongation zone-enriched portion; 4, maturation zone-enriched portion) is shown in the left panel. The right panel shows the increase or decrease (%) in relative transcript abundance obtained by deducing –N (%) to +N (%) values described in the left panel.

Independently from the absolute increases/decreases of transcript accumulation, by observing the percentage of mRNA distribution along the root, a widespread transcripts relocalization was clearly appreciable in the TZ-enriched portion upon nitrate provision for the majority of the upregulated genes (Fig. 7). Overall, 26 of the 35 analysed genes showed the maximum extent of transcription induction or repression in zone 2 of the root.

In a few cases, the increment observed in zone 2 was associated with a clear decrease in the percentage of mRNA abundance in all of the other three zones (for details, see Fig. 7).

### Tungstate and cPTIO affect the gene expression of selected genes differently

Previous results (Manoli *et al.*, 2014) allowed us to hypothesize a NO-dependent signalling pathway controlling the root growth response to nitrate.

To characterize better the putative role of NO signalling on the global transcriptomic response of maize root to nitrate, additional treatments with tungstate and cPTIO were performed and the level of expression of the 35 selected genes in the TZ-enriched zone was evaluated.

**Fig. 8.**
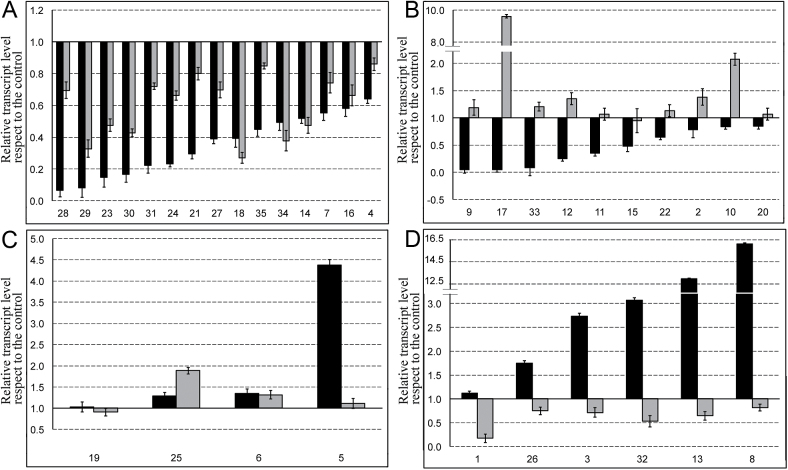
Expression profiles of the selected 35 genes in response to ${\text{NO}}_{3}^{-}$ , ${\text{NO}}_{3}^{-}$ +tungstate (W) and ${\text{NO}}_{3}^{-}$ +cPTIO treatments were assessed by real-time qRT-PCR. qRT-PCR results are reported as relative expression values according to the ratio between W/${\text{NO}}_{3}^{-}$ (black bars) and cPTIO/${\text{NO}}_{3}^{-}$ (grey bars). The expression levels were normalized against the maize *MEP* gene and the expression in the +${\text{NO}}_{3}^{-}$ TZ-enriched samples was set as 1 using the 2^–ΔΔ*C*^_T_ method. Error bars represent the SEM for three biological replicates. Genes were clustered according to their relative expression levels as: downregulated by both W and cPTIO treatments (A), downregulated by W but upregulated by cPTIO treatments (B), upregulated by both W and cPTIO treatments (C), and upregulated by W but downregulated by cPTIO treatments (D).

Overall, 25 of the 35 genes showed a significant decrease in transcription when tungstate was supplied together with nitrate (Fig. 8A, B). As tungstate is a nitrate reductase (NR) inhibitor, these results indicated that these genes do not respond directly to nitrate itself, but that their regulation relies on some nitrate assimilation products. Furthermore, among these genes, 15 were also clearly inhibited when a NO scavenger (cPTIO) was applied, allowing us to hypothesize a dependency of their transcription by the NO produced by NR.

The transcription of the 10 genes ranking in the other two subgroups (Fig. 8C, D) showed an increase in mRNA accumulation in the presence of tungstate, thus suggesting a direct regulation of their transcription by nitrate itself. However, six of these genes (Fig. 8D) also displayed a slight repression of their expression when cPTIO was supplied with nitrate. This seemed to indicate that genes grouped in Fig. 8D, besides being regulated by nitrate itself, were also induced by NO that was not derived from NR (which in this experiment was repressed by tungstate).

The promoter regions of genes ranking in the four groups A–D (corresponding to Fig. 8A–D, respectively) were screened for common *cis*-acting promoter elements, including transcription factor-binding sites, using Promzea (Liseron-Monfils *et al.*, 2013). *In silico* analysis of 1000bp upstream of the start codons of the co-regulated genes revealed that group-specific binding sites may be attributed to each group (Table 2). For example, genes belonging to group A shared octopine synthase gene (*OCS*) elements and *ARR1*, whereas *TGA1* was characteristic of group B transcripts. *ACE* and *LTRE* characterized the genes of group C, and *EIN3*, *EIL1*, and *ARF* binding sites were identified prevalently in the promoters of genes clustering in group D. Besides specific motifs, other shared transcription factor-binding sites, such as WRKY, ABRE, DRE, and MADS sites, were found in promoter regions of almost all members of the four gene clusters.

**Table 2.** Classification of the cis-elements represented in the promoter sequences of the four gene groups characterized by specific gene expression in response to cPTIO, tungstate, and ${\text{NO}}_{3}^{-}$ treatments Groups A–D correspond to the groupings in Fig. 8A–D, respectively.

*Cis*-element	Consensus sequence	Description	Group
ARRIAT	NGATT	ARR1-binding element	A
TELO-box	AAACCCTAA	Telomere motif	A
OCS	TGACGYAAGSRMTKACGYMM	Octopine synthase gene promoter element	A
HaHB-4		HD-Zip; JA and ET related	A
TGA1	TGACGTGG	HD-Zip	B
TEF-box	AGGGGCATAATGGTAA	Telomere motif	B
JASE2	CATACGTCGTCAA	A/C box-like motif	B
BLR-RPL-PNY	AAATTAAA	Homeodomain	B
TaNAC69	CCNAGGCACG	NAC domain	B
EIN3	GGATTCAAGGGGGCATGTATCTTGAATCC	Ethylene-responsive elements	D
EIL1	TTCAAGGGGGCATGTATCTTGAA	Ethylene-responsive elements	D
ARF	TGTCTC	Auxin response factors	D
PREATPRODH	ACTCATCCT	Pro- or hypo-osmolarity-responsive element (PRE)	D
ACEAtCHS	GACACGTAGA	ACE promoter motif	C
LTRE	ACCGACA	Putative low-temperature responsive element	C
LS7	TCTACGTCAC	bZIP	C
I-BOX	GATAAG	MYB, light regulation	C
DRE	TACCGACAT	Dehydration-responsive element	C
ATHB1	CAATTATTG	Homeodomain	C
W-box	TTGACT	WRKY	C

### Proteomic analysis

To complement the transcriptome study, a comparative iTRAQ-based proteome survey was performed on TZ-enriched segments after 2h treatments. Within this time frame, variations in a limited group of proteins were detectable, which indicated an early response to nitrate. In total, 880 accessions were identified by merging data obtained from two biological replicates (4179 unique peptides; Supplementary Table S5 at *JXB* online). The comparison between nitrate-supplied and nitrate-starved seedlings identified 107 differentially expressed proteins. Among these, 41 proteins were upregulated (fold change ≥1.3; Supplementary Table S5), five of which had a fold change of ≥1.5 (Table 3), and 65 were downregulated (fold change ≤0.77; Supplementary Table S5), 21 of which had a fold change of ≤0.67 (Table 3).

**Table 3.** iTRAQ differentially regulated proteins Proteins are indicated by the maize GDB accession ID. Fold changes indicate upregulated (≥1.5) and downregulated (≤0.7) proteins, according to the +N/–N treatment ratio. Accessions present in both RNA-Seq and iTRAQ lists are shown in bold.

Accession	Fold change	Description
*Glycolysis, gluconeogenesis, C-compound and carbohydrate metabolism*		
GRMZM5G828229	1.6	Dihydrolipoyl dehydrogenase
GRMZM2G440208	1.5	6-Phosphogluconate dehydrogenase
GRMZM2G166767	0.6	RHM1
*Nitrogen metabolism, amino acid metabolism and protein/peptide degradation*		
GRMZM2G102959	3.9	Ferredoxin–nitrite reductase
GRMZM2G050514	1.7	Glutamine synthetase root isozyme 1
*Cell defence*		
GRMZM2G051943	0.7	CHIA endochitinase A
GRMZM2G125893	0.7	Nucleoside diphosphate kinase
GRMZM2G373522	0.5	DHN-2 dehydrin
GRMZM2G108219	0.6	Peroxidase 11
GRMZM2G088765	0.5	Peroxidase 54
GRMZM2G044049	0.5	Similar to peroxidase
GRMZM2G427937	0.5	Peroxidase
*Post-transcriptional and post-translational mechanisms*		
GRMZM2G160994	1.7	PPR-like superfamily protein
AC233872.1	0.7	Mitochondrial glycoprotein
GRMZM2G464401	0.7	Plasminogen activator inhibitor 1 RNA-binding protein
GRMZM2G176707	0.6	Nucleosome/chromatin assembly factor group A
GRMZM2G045503	0.6	Similar to RNA-binding protein
GRMZM2G161746	0.6	Trypsin inhibitor heavy chain H3
GRMZM2G116282	0.6	Plasminogen activator inhibitor 1 RNA-binding protein
GRMZM2G157470	0.5	Brain acid soluble protein 1
*Cytoskeleton organization/vesicles trafficking*		
GRMZM2G071089	0.6	DREPP2
GRMZM2G001514	0.6	Fasciclin-like arabinogalactan protein 7
GRMZM2G137236	0.6	AP-2 complex, alpha subunit
GRMZM2G123558	0.7	DREPP4 protein
*Others*		
GRMZM2G109130	1.5	Lipoxygenase
GRMZM2G051270	1.5	ATP sulfurylase, sulfurylase 4, chloroplastic
GRMZM2G120876	1.5	Probable mitochondrial import receptor subunit TOM40-2
GRMZM2G358059	0.6	CRT1 calreticulin
GRMZM2G013461	0.6	CC4 multidomain cystatin

While the number of differentially expressed proteins was similar to that of nitrate-regulated transcripts, the overlap between changes in the proteome and transcriptome was remarkably small (20%). Out of 154 transcripts that changed in abundance after nitrate provision, only 21 of the encoded proteins were found to be differentially expressed.

**Fig. 9.**
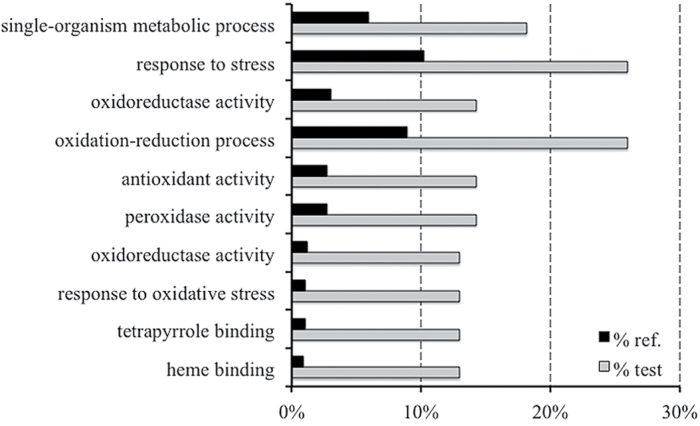
GO term enrichment analysis on proteome data obtained from iTRAQ analysis (Blast2GO software). The GO terms of ‘biological process’ and ‘molecular function’ were analysed, and significantly enriched categories (when compared with the entire proteome) (FDR≤0.05) were recorded. The percentage of over-represented GO terms among the iTRAQ differentially expressed proteins identified from the comparison between the TZ-enriched portion of seedlings grown in nitrate-supplied or -depleted solution are shown. Bars in grey indicate GO terms that were upregulated in the nitrate-supplied tissue versus the corresponding control (black bars).

To investigate further the relationship between modifications in transcript levels and proteins expression changes, a GO enrichment analysis was carried out on the iTRAQ-isolated proteins (Fig. 9). The results showed evident conservation with the RNA-Seq GO enrichment terms, showing that, in this case, a high number of GO terms were linked to oxidative stress (‘response to oxidative stress’, ‘oxidoreductase activity’, and ‘peroxidase activity’).

## Discussion

High-throughput sequencing approaches have become powerful tools to investigate the transcriptomes response to several abiotic stresses (Martin *et al.*, 2013). However, as also reported by Gifford *et al.* (2008), in most studies a number of transcripts risk being excluded because they might represent cell-specific transcripts whose expression is diluted when considering the whole tissue. In recent years, the precise transcriptomic analysis of specific cell types has demonstrated the importance of cell-specific components in the transcriptional response, which leads to diverse functional competence of the cells (Birnbaum *et al.*, 2003; Brady *et al.*, 2007; Gifford *et al.*, 2008). However, a precise transcriptomic analysis of the root TZ is still missing.

In this study, by using comparative Illumina-based transcriptomic and iTRAQ-based proteomic approaches, putative genes, proteins, and pathways that may be responsible for early events controlling nitrate sensing and signalling in the *Zea mays* L. root TZ were identified. Statistical analysis (*P*≤0.01, FDR≤0.05) revealed differential mRNA accumulation for a quite restricted group of transcripts (154 DEGs). As RNA-Seq allows a general high level of data reproducibility (Marioni *et al.*, 2008), the DEG selection was enlarged to accessions characterized by a less significant ratio between the two treatments (*P*≤0.01), to widen the range of information retrievable from the experimental design, avoiding the exclusion of some crucial component of this signalling. This enabled the identification of more than 600 genes putatively responsive to short-term nitrate treatment.

Most of the DEGs were upregulated, in accordance with other studies, conducted mainly on *Arabidopsis* and summarized in a recent review by Canales *et al.* (2014). The starvation pre-treatment probably turns off the expression of many genes related to nitrate assimilation, signalling, and transport, which are then switched on by the nitrate supply (Canales *et al.*, 2014).

The GO-enriched categories demonstrated did not include the most consistent GO terms identified by Canales *et al.* (2014), who analysed and integrated publicly available *Arabidopsis* root microarray data, probably because nitrate regulation of gene expression depends largely on the experimental context (Gutiérrez *et al.*, 2007; Krouk *et al.*, 2010*a*, b), and thus on the type of root cells analysed, confirming that cell sorting uncovers whole-genome responses to nitrate that are missed in whole-root studies (Gifford *et al.*, 2008). Moreover, functional annotations available for maize (48%) are less abundant and specific than those related to *Arabidopsis* (91%) (Yi *et al.*, 2013). However, considering the single annotations, a large set of genes (i.e. encoding LOB domain-containing proteins, glucose-6-phosphate dehydrogenase 3, urophorphyrin methylase 1, nitrite reductase 1, cytochrome P450, haemoglobin and nitrate transporter 2.1, and others) listed by Canales *et al.* (2014) as the top 50 most consistent and conserved genes in response to nitrate, was identified here, indicating a clear overlap between *Arabidopsis* and maize. Moreover, both proteomic and transcriptomic analyses recognized a number of ‘sentinel targets’ for the primary nitrate response such as ferredoxin and 6-phosphogluconate dehydrogenase (Medici and Krouk, 2014).

To support the RNA-Seq results, qRT-PCR validation was performed on arbitrarily chosen transcripts. A high degree (35 out of 41) of result reproducibility was recorded for transcripts selected among both the most significant ones (FDR≤0.05) and those ranking in the less strict group (*P*≤0.01), thus confirming the high reliability of the RNA-Seq approach. All showed a prevalent transcriptional regulation in the TZ-enriched root portion (zone 2), with some being more strongly (or exclusively) induced in this zone, while others also were more transversally regulated in other root portions (zones 1, 3, and 4). Overall, the results suggested the existence of individual and peculiar transcriptional profiles of the maize TZ cells in response to nitrate. This could derive from a different regulation of transcription, but also from a different time lapse of mRNA persistence, which might entail a broad relocalization of transcripts along the root.

Enriched biological processes related to the most significantly DEGs (FDR≤0.05) included GO terms associated with peroxidase activity. Peroxidases are encoded by a large multigenic family, and are involved in a wide range of physiological processes throughout the plant life cycle such as root elongation regulation through fine-tuning of the H_2_O_2_ level (Kawaoka *et al.*, 2003; Passardi *et al.*, 2005; 2006; Dunand *et al.*, 2007). Peroxidases are also part of the haem-binding proteins, which is the most representative enriched GO term. It comprises all proteins interacting selectively and non-covalently with haem, such as, beside peroxidases, nitrite and nitrate reductases, flavonoid 3-mono-oxygenases, cytochrome P450 superfamily proteins, and haemoglobins. In general, GO enrichment analysis showed that a short-term nitrate treatment induced the differential regulation of genes annotated as reactive oxygen species (ROS) related. A number of predicted oxidative stress response enzymes such as peroxidases were also identified by iTRAQ. These findings, as well as supporting the already known participation of ROS in nutritional responses (Shin *et al.*, 2005; Krapp *et al.*, 2011), also suggest that their localization in maize TZ cells might represent a crucial event for nitrate perception, as already demonstrated in the case of the response to hypoxia (Mugnai *et al.*, 2012),

To characterize further the potential physiological impact of the nitrate-regulated transcriptomic response, DEG sequence domains were classified against the InterPro database, revealing the presence of several groups of accessions such as transcription factors, membrane transporters, protein kinases, DREPP proteins, and, again, cytochrome P450 and peroxidases. Only a restricted group of transcription factors has been demonstrated to be implicated directly in regulating nitrate responses (Gutiérrez, 2012). Transcription factors known to regulate root development, cell proliferation, and elongation, such as ANR1 or HSP, MYB, ERF, and LOB domain members, were here demonstrated to be differentially regulated and specifically expressed in the TZ. LOB-domains affect meristem activity, organ identity, growth, and differentiation, and are required for PIN expression, thus regulating auxin transport in roots (Rast and Simon, 2012). Moreover, they have been reported to fine-tune the magnitude of the N response *in planta* by regulating a wide number of N-responsive genes and key transcripts for ${\text{NO}}_{3}^{-}$ assimilation (Rubin *et al.*, 2009; Yanagisawa, 2014).

The cytochromes P450 gene superfamily (*CYP*) encodes a large and diverse group of enzymes containing a haem co-factor. These haem proteins fulfil various biological functions through thousands of catalytic types, including reduction of NO to nitrous oxide and were demonstrated recently to participate in hormone biosynthesis. Three genes belonging to this family and orthologues of the *Arabidopsis Max1*, *Max3*, and *Max4* genes, which are involved in strigolactone (SL) biosynthesis (Zhang *et al.*, 2014; Umehara *et al.*, 2008; Gomez-Roldan *et al.*, 2008), were clearly downregulated by nitrate provision in the maize TZ-enriched segment. The same pattern of expression was observed for a gene encoding a PDR protein that may function as a cellular SL exporter facilitating delivery of SLs to their site of action (Kretzschmar *et al.*, 2012).

Furthermore, SL application was recently shown to reduce plasma membrane levels of PIN1 by enhancing clathrin-mediated endocytosis (Crawford *et al.*, 2010; Shinohara *et al.*, 2013). Proteome analyses performed here revealed the downregulation of AP-2, a protein involved in clathrin-mediated endocytosis for the regulation of indole-3-acetic acid (IAA) signalling and transport in plants (Yamaoka *et al.*, 2013). These results confirm the existence of overlaps between auxin and SL action (Cheng *et al.*, 2013) and the involvement of SLs in the nitrate response (Sun *et al.*, 2014; Yoneyama *et al.*, 2014), but also strengthen the previously hypothesized importance of TZ cells in early nitrate signalling in maize root (Manoli et al, 2014). Moreover, the identification of other components of auxin signalling, such as *AUX/IAA*, *SAUR* genes, POZ and TAZ domain-containing proteins, and an orthologue of *LCR69* or *LCR68*, corroborate the participation of this hormone in the root response to nitrate (Vidal *et al.*, 2010).

As well as auxin and SL, brassinosteroids (BRs) also seem to belong to the network of events involved in the adaptation to nitrate fluctuations. The induction of transcription of a gene encoding a BR receptor, *BRI1* (*Brassinosteroid-insensitive1*), demonstrated previously by Trevisan *et al.* (2011), was confirmed here. It showed a high extent of downregulation in the meristem and strong upregulation in the TZ, where it seemed to be induced in response to a nitrate assimilation product other than NO. Together with *BRI1*, the transcript amount of a *BRI1-Associated Receptor Kinase 1* (*BAK1*) also increased in the TZ of nitrate-supplied roots.

Furthermore, our data clearly confirmed the previously suggested involvement of NR and non-symbiotic-haemoglobins in early nitrate sensing by maize roots. Elhiti *et al.* (2013) suggested that class 2 non-symbiotic haemoglobins play a role in regulating the IAA synthesis and the PIN1-mediated transport of auxin by altering the level of NO in specific cells. This might suggest that auxin operates downstream of NO in the nitrate signalling in TZ cells of maize root.

NO is a bioactive molecule considered a general plant signal and is involved in an extremely wide range of physiological events in plant development, immunity, and environmental interactions (Yu *et al.*, 2014, and references therein). The production of NO by cells of the TZ also seems to be implicated in the pathway regulating the response to hypoxia in this same species (Mugnai *et al.*, 2012).

Here, after having confirmed the peculiarity of the transcriptional response to nitrate of the TZ cells, the putative involvement of NO in regulating gene expression was also examined further using tungstate and cPTIO, which inhibit NR activity and scavenge NO, respectively. The transcript levels of 25 of the 35 genes analysed were sensitive to tungstate, suggesting that their nitrate-induced regulation does not depend on nitrate itself but on some other nitrate assimilation products downstream of the NR activity. Among these, 15 (almost 50% of the randomly tested genes), which were also downregulated by cPTIO, seemed to be induced by NR-derived NO, thus allowing us to hypothesize a more generalized NO involvement in the pathway governing the nitrate response in TZ cells. The promoter regions of these NO-regulated genes share OCS and ARR1 *cis*-elements that were not identified in the other groups. OCS elements confer regulation by NO (Palmieri *et al.*, 2008), whereas ARR1 elements are typical cytokinin-responsive elements (Hwang *et al.*, 2012).

A more restricted group (10 of the 35 genes) was directly regulated by nitrate itself, although six of them also appeared to be NO responsive. Further experiments are needed to examine this odd result further.

The NO-mediated nitrate regulation of primary root elongation (Manoli *et al.*, 2014; Trevisan *et al.*, 2014) should depend on cytoskeletal rearrangements (Kasprowicz *et al.*, 2009). Several cytoskeletal genes were differentially regulated by nitrate supply in the TZ-enriched zone. These proteins participate in different plant processes, including establishing cell polarity, determining the location of the division plane, reprogramming of cell wall development and deposition, cell elongation, positioning receptors and transmembrane transport, transporting mRNAs within the cell, and positioning the nucleus (reviewed by Smith and Oppenheimer, 2005). Activation of the cytoskeleton was also demonstrated by the iTRAQ identification of vesicle-related and DREPP domain-containing proteins, a domain that confers microtubule-binding activity (Li *et al.*, 2011). Moreover, our data revealed that regulation of several targets related to cell wall deposition, modification, and reorganization was affected by nitrate supply, and probably results in altered root growth (Baluška *et al.*, 2003).

Nevertheless, it must be highlighted that, despite some fascinating information, only 20% of the protein profiles were supported by the transcriptomic data. A generally low congruency of proteomic and transcriptional profiles has been reported previously (reviewed by Vogel and Marcotte, 2012). Several factors may cause the small overlap between the two approaches. First, iTRAQ technology might not detect proteins with a low abundance, while RNA-Seq could detect low transcript levels of the corresponding genes (Lan *et al.*, 2012). Moreover, a change in transcript abundance may not be rapidly translated into changes in protein level (Rajasundaram *et al.*, 2014), suggesting the existence of a lag time between ${\text{NO}}_{3}^{-}$ -induced transcription and translation. Furthermore, proteins may be synthesized in a specific tissue and then move towards another, as in the case of UPB1, which is implicated in the control of ROS homeostasis along *Arabidopsis* roots (Tsukagoshi *et al.*, 2010). Here, transcription of a gene encoding a maize orthologue of UPB1 was upregulated by nitrate even when the protein was not included in the iTRAQ output in the same root zone. Furthermore, the existence of microRNA-guided post-transcriptional mechanisms of regulation must be considered. In fact, *in situ* hybridization of transcripts for nitrate-responsive maize microRNA revealed their localization in cells of the meristem and TZ (Trevisan *et al.*, 2012*a*, *b*).

Before concluding, it must be highlighted that 13% of the upregulated DEGs were transposable elements. Several studies have reported transposable element overexpression following abiotic or biotic stress in plants (reviewed by Benoît Chénais *et al.*, 2012). Our findings endorse the important role of epigenetic processes in adaptation to nitrate fluctuations.

**Fig. 10.**
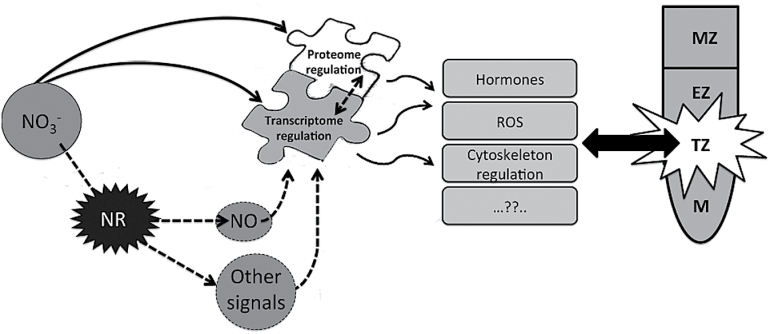
Proposed model for the nitrate response in the maize root TZ. After nitrate supply, the transcription of a wide set of genes is regulated. This reprogramming in the transcriptome could be translated as a proteome rearrangement. The transcriptome change could be dependent on both nitrate itself or on some NR-derived product, such as NO, which was confirmed to represent a key signal in the root nitrate response. These molecular events could be implicated in the physiological adaptation of plants to nitrate fluctuations in soil. MZ, maturation zone; EZ, elongation zone-enriched portion; TZ, TZ-enriched portion; M, meristem-enriched zone.

To conclude, information obtained from the two high-resolution data sets depicted a snapshot of genes and proteins participating in the early response to nitrate in the root TZ (Fig. 10). The identification of candidate genes differentially regulated by the presence of nitrate and located specifically in this root zone demonstrated the spatial transcriptional complexity that underlies the root response to nutritional inputs. The TZ was confirmed as a critical zone in sensing nitrate, which seems to directly influence the transcript levels for a few genes but also to act indirectly through the NR action. NO was definitely established as a key player in the maize root response to nitrate, but other nitrate-derived signals also seem to contribute to this pathway. Both transcriptomic and proteomic approaches suggested that ROS signalling might play a pivotal role in the complex signalling featuring nitrate perception and leading to root development tuning, probably controlling the balance between cell proliferation and cell elongation and thus accomplishing the developmental plasticity that typically characterizes this root zone.

## Supplementary data

Supplementary data are available at *JXB* online.

Supplementary Table S1. Primers sequences for the selected genes tested in qPCR validations.

Supplementary Table S2. RNA-Seq dataset and DEGs information.

Supplementary Table S3. InterPro domains of DEGs identified by RNA-Seq analysis.

Supplementary Table S4. List of 41 selected DEGs and RT-PCR variations.

Supplementary Table S5. iTRAQ dataset.

Supplementary Data

## Abbreviations:

BR: brassinosteroid
cPTIO: 2-(4-carboxyphenyl)- 4,4,5,5-tetramethylimidazoline-1-oxyl-3-oxide
DEGs: differentially expressed genes
FDR: false discovery rate
GO: Gene Ontology
IAA: indole-3-acetic acid
iTRAQ: isobaric tags for relative and absolute quantitation
LC-MS/MS: liquid chromatography tandem mass spectrometry
N: nitrogen
NO: nitric oxide
NR: nitrate reductase
qRT-PCR: quantitative reverse transcription PCR
RNA-Seq: RNA sequencing
RPKM: reads per kb per million
ROS: reactive oxygen species
SL: strigolactone
TZ: transition zone.
