# Supplementary material for: Nitrate sensing by the maize root apex transition zone: a merged transcriptomic and proteomic survey
Source: J Exp Bot. 2015 Apr 23;66(13):3699–715. doi: 10.1093/jxb/erv165 (PMC4473975; doi:10.1093/jxb/erv165)
Supplement: Supplementary Data [file supp_66_13_3699__index.html]

Nitrate sensing by the maize root apex transition zone: a merged transcriptomic and proteomic survey — Nitrate sensing by the maize root apex transition zone: a merged transcriptomic and proteomic survey — Supplementary Data 

# Nitrate sensing by the maize root apex transition zone: a merged transcriptomic and proteomic survey

## Supplementary Data

Data files

**Files in this Data Supplement:**

- Supplementary Data - Supplementary Data
- Supplementary Data - Supplementary Data
