# Supplementary material for: Nitrate sensing by the maize root apex transition zone: a merged transcriptomic and proteomic survey
Source: J Exp Bot. 2015 Apr 23;66(13):3699–715. doi: 10.1093/jxb/erv165 (PMC4473975; doi:10.1093/jxb/erv165)
Supplement: Supplementary Data [file supp_erv165_jexbot143826_file001.pdf]

Nitrate sensing by maize root apex transition zone: a merged transcriptomic and proteomic survey

Sara Trevisan, Alessandro Manoli, Laura Ravazzolo, Alessandro Botton, Micaela Pivato, Antonio Masi, and Silvia Quaggiotti

Supplemental Tables S1 (qPCR Primer list) and S4b (qPCR Validation Dataset)

Supplementary Table S1. List of primers used in Real Time qPCR analyses.

Primer	**Fw Sequence 5' to 3'**	**Rev Sequence 5' to 3'**
**GRMZM2G035370_T01**	ACGGCAGGAGGAGGAAGAT	GTCAAACCCTACGATTACAAAGC
**GRMZM2G145914_T01**	GGTACAACAAGCACCTTCCG	AACTCCGCTAAACCGCTACT
**GRMZM2G001205**	GACTGCCTTGGCTACTAGATAG	CCTCCGCCCAGAAGCAAAT
**GRMZM2G029518**	CCGCATTAGTGTCAGTCCATTC	CCGGATGCAACGACCTTAAC
**GRMZM2G125482**	AGGAGGAGTTCAGCATGGAG	CGGCAACAACAGGAGCAATA
**GRMZM2G046532**	AGCCAACACACACACAAATACT	TTCCTCGCCGACACCATAG
**GRMZM2G106190**	CCTGCATCTCCTACCCCAAG	AGCCCATCTCAACAACGAAAC
**GRMZM2G179294**	CGATGAGTGGCTGTCGTTG	TTGAGCCCCTCCATTCGTAA
**GRMZM2G080183**	TCCGTCCATTCATCCATCCA	CCGTGGCATTCATTTACATTGG
**GRMZM2G036629**	CTGGTCTGACTCTGGCTCTC	AGCCAATCGTTACAGGGACA
**GRMZM2G112782_T01**	TGACTGGTGGTGTTCTCTCG	CCTCCTTTGGCAGCAATCAG
**GRMZM2G340444**	GAGAACAAATCACCCCAGTCA	AGGTCGCAAATGAGGAAAGC
**GRMZM5G856929**	GCCACGTCGTCTGGGAAT	AGAATCAAACTCTCTGCAAGGA
**GRMZM2G008353**	GTTTTGGCATCTGTCACCGT	AGCGGCACAACATATGAACA
**GRMZM2G044902**	GCCAAACCAAACCAAACCAC	TACCGCTACTACGCAACCAA
**GRMZM2G021427**	CTACTGCGCTCTGATTCCCT	GCCTTGGTCACACTGATTCA
**GRMZM2G146004**	AGGTGTTCTGTCGTCTTGGT	TGCTCTGCTCTGCTCTGAAT
**GRMZM2G004161_T04**	CCAACAGGAAGCAGATGAGC	CGTAGGGTGACAAAGTAGGC
**GRMZM2G118951**	CCCAGGCATTTTCTTTTCCTTT	AATTGCCGCCACTCAACG
**GRMZM2G177077**	CACTATCTCGCCGCCAAGTA	TGCCCGCCTTATTCTCATCT
**GRMZM2G058760**	GCCCAGCTCAAGAAGAACAA	CTTTACACCGCCCCGAGAA
**GRMZM2G105604_T01**	AGTGGTATCCTTGTCACCCT	GTCGAACCTGTGCTCCCATA
**GRMZM2G366659**	ATTACGTGGACTAGGCACCA	CGAAACGGCGAACTTCAACT
**GRMZM2G009223**	TCATCGTCGCGTCCATCAT	TTCGGCGGTGAACTGAACT
**GRMZM2G440208**	AGGGATGTTTTCTGCTTTGTGT	CATTGGAGTTTGAGAGGCATCA
**GRMZM2G105348**	GTTCGGCAGCGATTACCTG	CCACTTCAGTCCAACACGTC
**GRMZM2G124495**	CGAAGATAAGCTAGCCTGTCG	CCTCCAAAACACTCGTCGTC
**GRMZM2G099642**	AGCTGGCGTAGAAAGAGAAC	CCCACTACCCCAATAAAGCC
**GRMZM2G181081_T04**	CACTTGGCTGCGTGTAGGA	GAATGTGCCTTCGCCTATGG
**GRMZM2G167520_T01**	TAGGGAAGGCAAGGCAAGG	TGAATGCACATCTGAACCTGT
**GRMZM2G143469**	GCTAACAGAGTACGCAACAGG	CCAAACAGGGCAGCACAAA
**GRMZM2G134432**	GAGGCGGCTCAAATCAGAC	AGGGTTGTTTACGAGACAGGA
**GRMZM2G070603**	GCAGGACCAGGCTGTTACA	TGCTGATGGACGATGGATCT
**GRMZM2G166082_T02**	AGCAGGCTGAGGAATGTGAA	TCAAACATCACGCAAACCTGT
**GRMZM2G145008**	CGTCTTGGACTTGGCATGTT	CCCGGTAGTGCTGAATAATCA
**GRMZM2G168898**	GGCTGTTGATGCTTCCTAGC	ATGACGGGCCTTTTCTGAAT
**GRMZM2G079381_T02**	CTTCA TGGGCTGCCTCAC	CGCTTGACGAAGGTCCTACT
**GRMZM2G067402_T02**	GGAGCCTCGAGA TGAAGAAA	ACAATACACGCTCCCTCCAG
**GRMZM2G568636**	ATGATCCAGTTCGCCATCTC	GTCCGTGGTACGTCGTAGGT
**GRMZM2G015933**	TCCTCTCCCTTGTCGTTGTT	AGCCTTGA TCCAGGACTCTTC
**GRMZM2G010251**	GAGAAGAGCAAGGGACTCCA	CTCA TGTCAACGGAGCACAC

Supplementary Table S4**.** List of 41 selected DEGs obtained from RNAseq analysis carried out on zone 2 of nitrate starved seedlings after 2h of nitrate supply (+N) or depletion (-N). DEGs were divided into up-regulated or down regulated according to their +N/-N RPKM ratios. DEGs which expression profiles were not confirmed by Real time PCR were evidenced in grey.
no*= p ≤ 0.01, FDR ≤0.05; no**= FDR>0.05.

**Gene ID**	**Description**	**-N**	**+N**	**Significant**	**Orthologs**
**Up-regulated**					
GRMZM2G168898	Non-symbiotic hemoglobin 2, *Hb2*	0.02	50.63	yes	AT2G16060; *GLB1* hemoglobin 1
GRMZM2G029518	Fibroin heavy chain	0.04	8.33	yes	LOC_Os10g31540.1, glycine-rich cell wall structural protein 2 precursor (*Oryza sativa*)
GRMZM2G124495	Putative MYB DNA-binding domain superfamily protein	0.04	5.36	yes	AT5G06800; myb-like HTH transcriptional regulator
GRMZM2G143469	One-helix protein 2, *OHP2*	0.04	5.33	yes	AT1G34000; *OHP2* one-helix protein 2
GRMZM2G105604_T01	Uncharacterized protein, Uroporphiryn-III C-methyltransferase conserved site	2.56	108.95	yes	AT5G40850.1*; UPM1*, urophorphyrin methylase 1
GRMZM2G004161_T04	Uncharacterized protein, TAZ-type domain	3.36	97.19	yes	AT3G48360; *BT2*, BTB and TAZ domain protein 2
GRMZM2G146004	Uncharacterized protein, LEA-5 protein domain	4.55	129.39	yes	AT4G02380.1; AtLEA5
GRMZM2G079381_T02	Ferredoxin-nitrite reductase, *NiR*	3.03	81.53	yes	AT2G15620; *NIR1*, nitrite reductase 1
GRMZM2G067402_T02	Non-symbiotic hemoglobin, *Hb*	39.31	930.71	yes	AT2G16060; *GLB1*, hemoglobin 1
GRMZM2G106190	*FDX6*	34.99	806.8	yes	AT5G10000; *ATFD4*, ferredoxin 4
GRMZM2G058760	Uncharacterized protein, Ferredoxin reductase-type FAD-binding domain	35.93	653.95	yes	AT1G30510; *ATRFNR2*
GRMZM2G177077	Glucose-6-phosphate 1-dehydrogenase	8.62	156.22	yes	AT5G13110; *G6PD2*
GRMZM2G035370_T01	Myb domain	0.18	3.12	no	AT5G06800; myb-like HTH transcriptional regulator
GRMZM2G009223	Glucose-6-phosphate/phosphate translocator 2	0.47	7.31	yes	AT1G61800; *GPT2*
GRMZM2G145008	Uncharacterized protein, ArfGap/RecO-like zinc finger protein domain	0.03	0.4	no*	AT1G08680; *ZIGA4*, ARF GAP-like zinc finger-containing protein
GRMZM2G440208	6-phosphogluconate dehydrogenase, decarboxylating	34.28	438.05	yes	AT1G64190; 6-phosphogluconate dehydrogenase
GRMZM2G181081_T04	CIPK-like protein 1	1.15	13.8	yes	AT2G26980; *CIPK3*, CBL-interacting protein kinase 3
GRMZM2G145914_T01	Uncharacterized protein, Oxoglutarate/iron-dependent dioxygenase domain	2.65	24.93	yes	AT2G48080; oxidoreductase, 2OG-Fe(II) oxygenase
GRMZM2G568636	Nitrate reductase (NADH), *NR1*	1.23	9.05	no *	AT1G37130; *NIA2*, nitrate reductase 2
GRMZM2G167520_T01	Brittle stalk-2-like protein 6, COBRA-Like domain	1.13	7.59	yes	AT3G02210; *COBL1*, COBRA-like protein 1 precursor
GRMZM2G015933	BRASSINOSTEROID INSENSITIVE 1-associated receptor kinase 1, *BRI1*	0	0.07	no**	AT1G71830.1; *ATSERK1*, somatic embryogenesis receptor-like kinase 1
GRMZM2G366659	Putative trehalose phosphatase/synthase family protein	5.24	31.18	yes	AT1G23870; *ATTPS9*
GRMZM5G856929	Uncharacterized protein, Galactose oxidase central domain	1.65	8.95	yes	AT1G31350; *KUF1*
GRMZM2G046532	Flower-specific gamma-thionin	2.5	13.11	no*	AT2G02130.1; *LCR68*
GRMZM2G044902_T03	Uncharacterized protein, LOB domain	8.22	39.42	yes	AT5G67420.1; *LBD37*

GRMZM2G179294	*NAR2.1*	67.91	322.89	yes	AT5G50200.1; *ATNRT3.1*
GRMZM2G001205	ZFP16-1, Zinc finger, C2H2-like domain	8.54	40.45	yes	AT5G59820.1; *RHL41*,ZAT12, C2H2-type zinc finger
GRMZM2G008353	Uncharacterized protein, Phosphate-induced protein 1 domain	40.5	174.11	yes	AT4G08950.1; *EXO*
GRMZM2G010251	High affinity nitrate transporter, *NRT2.1*	0.97	4.1	yes	AT5G60770.1; *NRT2.4*
GRMZM2G340444_T01	Putative RING zinc finger domain superfamily protein	2.1	8.43	no*	AT4G40070.1; RING/U-box superfamily protein
GRMZM2G080183	Uncharacterized protein, Haem peroxidase domain	21.07	74.19	no*	AT2G18980.1; Peroxidase superfamily protein
GRMZM2G036629	Metallothionein-like protein 1	140.87	417.25	no*	AT3G09390.1*; ATMT-1*, metallothionein 2A
GRMZM2G166082_T02	Unknown protein	1.1	2.86	no**	AT2G16270.1; unknown protein
**Down-regulated**					
GRMZM2G118951	NDR1/HIN1-Like protein 2	136.67	57.31	no**	AT1G17620.1; Late embryogenesis abundant (LEA) hydroxyproline-rich glycoprotein family
GRMZM2G112782_T01	Uncharacterized protein, SNARE protein domain	29.38	10.55	no*	AT4G30240.1; Syntaxin/t-SNARE family protein
GRMZM2G125482	Flavonoid 3-monooxygenase	21.29	6.32	yes	AT5G07990.1; CYP75B1, Cytochrome P450 superfamily
GRMZM2G021427	Uncharacterized protein, RlpA-like double-psi beta-barrel domain	32.17	7.15	yes	AT1G65680.1*; ATEXPB2*
GRMZM2G070603	Peroxidase 1	7.1	1.57	yes	AT1G05260.1; *RCI3*, Peroxidase superfamily
GRMZM2G105348	Uncharacterized protein, Heat shock factor (HSF)-type	6.34	1.2	yes	AT3G24520.1*; AT-HSFC1* w
GRMZM2G099642	Putative alcohol dehydrogenase superfamily protein	9.03	0.93	yes	AT5G17000.1; Zinc-binding dehydrogenase
GRMZM2G134432	Uncharacterized protein, PK-like domain	1.43	0.14	yes	AT2G19130.1; S-locus lectin protein
